# E-Learning as a Factor Optimizing the Amount of Work Time Devoted to Preparing an Exam for Medical Program Students during the COVID-19 Epidemic Situation

**DOI:** 10.3390/healthcare9091147

**Published:** 2021-09-02

**Authors:** Magdalena Roszak, Bartosz Sawik, Jacek Stańdo, Ewa Baum

**Affiliations:** 1 Department of Computer Science and Statistics, Poznan University of Medical Sciences, 60-806 Poznan, Poland; mmr@ump.edu.pl; 2 Department of Business Informatics and Engineering Management, AGH University of Science and Technology, 30-059 Krakow, Poland; 3 Haas School of Business, University of California at Berkeley, Berkeley, CA 94720, USA; 4 Department of Statistics, Computer Science and Mathematics, Public University of Navarra, 31006 Pamplona, Spain; 5 Centre of Mathematics and Physics, Lodz University of Technology, 90-924 Lodz, Poland; jacek.stando@p.lodz.pl; 6 Department of Social Sciences and the Humanities, Poznan University of Medical Sciences, 60-806 Poznan, Poland; ebaum@ump.edu.pl

**Keywords:** e-learning, digital training, healthcare education, innovation in teaching, clinical teaching, e-exams

## Abstract

The COVID-19 pandemic had a huge impact on the learning and teaching processes, particularly in healthcare education and training, because of the principal position of the cutting-edge student–patient interaction. Replacing the traditional form of organization and implementation of knowledge evaluation with its web-based equivalent on an e-learning platform optimizes the whole didactic process not only for the unit carrying it out but, above all, for students. This research is focused on the effectiveness of the application of e-learning for computer-based knowledge evaluation and optimizing exam administration for students of medical sciences. The proposed approach is considered in two categories: from the perspective of the providers of the evaluation process, that is, the teaching unit; and the recipients of the evaluation process, that is, the students.

## 1. Introduction

During the COVID-19 pandemic and mandatory lockdown, academic institutions have shifted to distance learning. This pandemic had a massive impact on the learning and teaching processes, especially in healthcare education, due to the predominant role of the current student–patient interaction. Replacing the traditional form of organization and implementation of knowledge evaluation with its web-based equivalent on an e-learning platform optimizes the whole didactic process not only for the unit carrying it out but, above all, for students. This research is focused on the effectiveness of the application of e-learning for computer-based knowledge evaluation and optimizing exam administration for students of medical sciences. The proposed approach is considered in two categories: from the perspective of the providers of the evaluation process, that is, the teaching unit; and the recipients of the evaluation process, that is, the students. Worldwide higher education institutions were forced to accelerate the introduction of web-based learning methodologies in areas where this was not the main core, such as clinical teaching. This paper presents the current trends and new challenges that emerge from this new e-learning environment, focusing on its potential to revolutionize healthcare education and exploring how it may help to better prepare future healthcare professionals for their daily practice. The process of optimization through e-learning should become a natural part of the didactic process conducted in every subject at all types of higher education institutions, including medical universities.

For more than a decade, medical schools have been working to transform pedagogy by eliminating/reducing lectures; using technology to replace/enhance anatomy and laboratories; implementing team-facilitated, active, and self-directed learning; and promoting individualized and interprofessional education [1,2,3]. The situation of the spread of the novel Severe Acute Respiratory Syndrome Coronavirus 2 (SARS-CoV-2) accelerated the application of online teaching and examination in medical schools around the world. Many authors recently considered these issues, for instance, as in a recently published paper by Bianchi et al. about the effects of the SARS-CoV-2 pandemic on medical education [4]. Bianchi et al. presented considerations and tips for the Italian medical education system under the new circumstances of COVID-19 [4]. While the COVID-19 outbreak has been one of the most significant challenges faced in the history of medical education, it has also provided an impetus to develop innovative teaching practices, bringing unprecedented success in allowing for medical students to continue their education, for instance, in ophthalmology, despite these challenges [5]. Different types of online courses are provided at present to develop and implement an effective learning process for medical students. Paper [3], by Rose, presents and discusses the challenges in medical students’ education in the time of COVID-19. This author also pointed out that additional unknown academic issues will require attention, including standardized examinations when testing centers are closed, the timeline for residency applications for current third-year students, and the ability to meet the requirements for certain subspecialties prior to applying to residency [3]. Another interesting example of the educational challenges during this pandemic can be found in medical and surgical nursing, with a core course in baccalaureate nursing programs that requires active and effective teaching and learning strategies to enhance students’ engagement [6]. The unprecedented, abrupt shift to remote online learning within the context of the national lockdown due to the 2019 coronavirus disease (COVID-19) highlights the importance of addressing students’ preparedness in managing their first experiences with online learning [7]. Many authors have tried to explore the medical students’ and faculty members’ perspectives of online learning during the COVID-19 era [8]. As examples, two recent papers by Varvara et al. [9] and by Iurcov et al. [10] consider the impact of this pandemic on academic activity and health status among medical dentistry students in Romania [9], and also in general dental education challenges during COVID-19 for dentistry undergraduate students in Italy [10]. At present, during the COVID-19 pandemic, e-learning has become a potential approach to technology in education that provides contemporary learners with authentic knowledge acquisitions. As a practical contribution, electronic examination (e-exam) is a novel approach to e-learning, designed to solve traditional examination issues. It is a combination of assorted questions designed by specialized software to detect an individual’s performance. Despite the intensive research carried out in this area, the completion of e-exams brings challenges, such as authentication of the examinee’s identity and answered papers [11]. It is important to explore the factors affecting students’ preference for remote e-exams, methods of course assessment/evaluation, factors related to students’ exam dishonesty/misconduct during remote e-exams and measures that can be considered to reduce this behavior [12]. This type of research has been carried out in many medical schools around the world to evaluate the experience of students at faculties of Medicine, Dentistry, Pharmacy, Nursing and Applied Medical Sciences regarding remote e-exams preferences and academic dishonesty during the pandemic [12].

## 2. Technologies in Education

Technology has always changed methods of learning and knowledge transfer. Generalized access to the Internet has brought about a revolution in learning and teaching. In one new method, a technologically new way of publishing educational content, we now have previously known methods along with new elements that had no equivalent in the past [13,14,15,16]. Their emergence in education is determined by the application of modern digital technologies of sound and image recording and their integration with traditional text-based instruction [17,18]. The instant sharing of e-materials for education participants and their prompt updating by teachers is also of crucial significance. The evaluation of student knowledge and the learning process has also been revolutionized [19]. Online tests including a broad interface of questions and automatic verification of answers are now available, as well as self-study tests with explanations and decision-making labyrinths which encourage creative thinking [20,21,22]. The authors of papers [23,24,25,26] have published very interesting recent examples of the usage of technological innovation in medicine. In paper [23] by Guiter et al., the authors present the development of remote online collaborative medical school pathology and explain how students across several international sites, throughout the COVID-19 pandemic, could control the digital slides and offer their own diagnoses, followed by group discussions. In publication [24] by Guadalajara et al., the authors demonstrate whether it is possible to create a technological solution to flexibly self-manage undergraduate general surgery practices within hospitals. In this interesting research study, it was proven that the usage of innovative educational technology could be efficient. The use of mobile-learning application designed to be an educational opportunities’ manager tool might be very helpful in promoting self-directed learning, flexible teaching, and bidirectional assessments. The authors also show some limitations for teachers who employ a personal teaching style, which may not need either checkerboards or a tool. Presented solution [24] supports teaching at hospitals in a pandemic without checkerboards. In paper [25], by Bianchi et al., the authors concentrated on an evaluation of the effectiveness of digital technologies during anatomy learning in nursing school. Nicholson et al. also considered anatomy in paper [26], but as an interactive, multi-modal anatomy workshop. The authors proved that an interactive workshop improved attendees’ examination performance and promoted engaged enquiry and deeper learning. This tool accommodates varied learning styles and improves self-confidence, which may be a valuable supplement to traditional anatomy teaching [26].

## 3. Changes and Challenges in the Process of Education at an Academic Level

At present, students take full advantage of new digital technologies in both their daily lives and in the process of formal and informal education [27,28]. In higher education institutions, where teaching and learning are pursued only in the form of traditional practical and laboratory classes held in classrooms, learners do not find the means of information transfer that they know from the Internet. This contributes to a decrease in effective memorizing and generally reduced motivation to learn [29,30,31]. Distance education is a new method of working with students, which is becoming more crucial in current academic education, particularly in the face of the COVID-19 epidemic [32,33,34].

Technological progress inevitably leads to the implementation of up-to-date technologies in distance education at every level, including continuing education [17,35,36,37]. Students particularly favour interactive online courses, as they seem to produce better effects than traditional methods in terms of knowledge acquisition [38,39,40,41]. The numerous advantages that online learning offers leads students to turn to the Internet and multimedia sources of knowledge more than they turn to traditional textbooks [42,43]. Therefore, it seems appropriate to implement online learning and provide access to multimedia materials that include reliable educational content, which can replace traditional classes. The application of the methods and techniques of distance learning can be a source of competitive advantage for the school. It can considerably contribute to the quality and efficiency of contemporary student education [44,45].

The methods and techniques of distance education are commonly applied in medical schools, and scientific reports confirm their comparable or even greater effectiveness in comparison with traditional forms [16,37,38,44]. Acquiring skills in virtual reality will translate to a higher quality of medical procedures being administered to patients.

To date, most online trainings were intended for doctors (58%) and also for nurses, pharmacists and stomatologists [46]. It should be remembered that e-learning is not exclusive to higher education [47,48,49] but is also used for courses or training that the future graduates of medical schools will attend, broadening their knowledge after their medical studies [50]. A lack of experience with participating in online education is a burden for a graduate, as there is no easy way to gain such skills outside of school. Thus, distance learning allows the students to develop additional competences; not just digital ones but also soft competences [51]. All of this facilitates the development of skills such as collaborative work, time management and problem-solving, as well as encouraging creativity and flexibility. Consequently, embracing state-of-the-art technologies may result in a growing number of better-educated graduates who can adapt to the changing labour market and are interested in positions that present nontypical professional and scientific challenges.

Before the pandemic, distance education in medical universities, due to its character, was pursued mainly as part of a hybrid system, in which part of the learning process takes place in direct contact with the teacher in the classroom, and the other part takes place online [39,40,48]. In the case of practical classes, virtual education is combined with supervised hands-on practice, performed on patients in a hospital or medical simulation centres. Such is the nature of most courses taught at medical universities.

Additionally, education through simulation is becoming increasingly popular in the medical academic environment. This is the best teaching method, enabling the creation of real situations in risk-free conditions. Decision-making games can be used successfully in the educational process of future medical staff. The aim of this work was to create a didactic computer program “Trauma”, analyze its impact on students’ knowledge of the direction of medical rescue and evaluate the attractiveness of classes conducted using this method. The results show that the use of the “Trauma” program in didactics has allowed for improvements in the knowledge and skill levels of students taking part in the study in the field of trauma patients’ treatment. In the assessment of students, the classes in which the program was used were interesting. The vast majority of respondents would like to participate in such classes again [52].

There are few reports concerning distance education in medical schools in Poland, especially its application in teaching and learning, as well as in evaluation, comprising credits and examinations [19,20,21,39,40,53]. It can, therefore, be concluded that, prior to the outbreak of the COVID-19 pandemic, distance learning was not common or its scope was limited. The present paper is a contribution to an academic discussion on e-learning for basic sciences and particularly its use to optimize the amount of time devoted to preparing an exam for medical programs. It looks at two categories: its usefulness for the educational institution and the recipients of the process—in this case, medical university students. The scope of the article involves a comparison of the amount of work time is needed before an examination administered entirely in a computer-based form and how much work time is needed before one given in a traditional paper-based form.

## 4. Data and Methods

The data collected in the article present the results of research into the working time and organization of electronic (in an e-learning portal) and traditional version of the evaluation of medical knowledge (without e-learning technologies) expressed in minutes (clock hours). The statistical analysis is based on descriptive statistics with the use of Excel [54] and R language [55]. The analysis shows the sum of the working time (clock hours) according to own formulas describing the same process, including a way to build databases for knowledge evaluation. There was no need to statistically analyze the collected data using statistical tests, as the complete data were compared.

The research conducted by the authors is very demanding, due to the labor-intensive preparation of databases, level of technology competence, slower implementation of e-learning in medical education, which is significantly different from education in other fields, and its use by a large group of students from one year. For this reason, standard educational theories were insufficient and could not fully meet all the conditions for the study and the stated goal of the authors’ analyses. It was necessary to develop own procedures, accounting for the e-learning methodology.

The research necessary to carry out an analysis of the optimization of knowledge evaluation was conducted in the Department of Pathophysiology in cooperation with the Department of Computer Science and Statistics of Poznan University of Medical Sciences, Poland (PUMS) in the academic years of 2009–2019. Academic teachers (knowledge supply), technical and administrative staff (organizational support) and medical e-learning experts were involved in this research. An electronic evaluation of knowledge was conducted on the Online Learning and Training (OLAT) e-learning portal under an open source license.

The analysis of working time and the organization of electronic knowledge evaluation was carried out on 333 students in their second year of medical studies in the preclinical subject of pathophysiology in the 2018–2019 academic year. These students were studying for one year, and completed the subject at the same time.

The analysis of teachers’ working time in the preparation of substantive content in pathophysiology, including the database of international standards for testing and assessment [19,20], the Question and Test Interoperability (QTI), was carried out in the 2015–2016 academic year. The same team of teachers and employees supporting the work participated in the organization and implementation of the evaluation of the delivered traditionally and with the use of an e-learning portal. It was necessary to compare both versions, implementing the evaluation of knowledge in pathophysiology for a large group of students at the same time.

## 5. Preliminary Conditions for the Analysis of Work Time Devoted to Administering Examinations in Medical Sciences

The analysis of the amount of time devoted to the evaluation of students’ performance requires data on the distribution of tasks necessary to set regular exam and paper-based credit test in comparison with its electronic counterpart on an e-learning platform. The organization of the evaluation of students’ knowledge usually involves teachers, supported by administrative and/or technical-engineering staff. Student evaluation, conducted in large groups in the traditional form, requires cooperation between these staff members in the auditorium hall or a lecture hall in which the exam or credit test is held; their work is purely organizational, not substantive.

The analysis of the amount of time devoted to the evaluation of students’ knowledge in the medical program was conducted using the subject of pathophysiology as an example. This subject is taught in the 2nd year of studies, where the number of students is very high and often varies between 200 and 400. This serves to demonstrate the usefulness of e-learning for the evaluation of students’ knowledge. The example presents how it is carried out on an e-learning platform, which complies with the international standards for testing and assessment [19,20], Question and Test Interoperability (QTI).

The study was conducted on a sample of one grade-level group, comprising 333 students taking the course over one semester of an academic year at the Department of Pathophysiology in PUMS [53].

## 6. Implementation of Evaluation of Knowledge in Pathophysiology

Continuous assessment tests (benchmark tests) given throughout the course, as well as the final test, had to be administered on the premises of the university, according to the act on studies pre-COVID [56].

The course in pathophysiology includes three tests:

1. An introductory test in physiology, beginning the subject;
2. A test on clinical cases, summarizing practical classes;
3. A final test covering the substantive knowledge provided during practical courses and seminars.

Lectures in the subject also end with a summative test, which constitutes either part of the final exam or the credit required for course completion.

The analysis of the process of knowledge evaluation does not account for retakes (two additional attempts) to which students who fail continuous assessment are entitled. Similarly, the report does not consider retake final exams, which are available to people who fail the final exam. Sometimes a need arises to organize a committee course crediting for students who do not pass the course according to the above rules, or a committee exam granted by the Dean, following the consideration of an individual application submitted by the student or teacher.

Following the School Regulations, there must be two dates for the exam, settled by the student representative and the examiner. Additionally, the so-called pre-term exam date may be established, which increases the number of exam dates. Consequently, the minimum number of dates scheduled for the first-attempt examination is three. Every student independently decides on which day he or she wants to take the exam, considering his or her credit and exam calendar. Still, other individual cases have to be noted when a student or a group of students requests a different date to those already scheduled, which may be due to a fortuitous event or the individual organization of studies. As is apparent from the above, the evaluation of knowledge is somewhat burdensome for the unit responsible for the teaching process and the exam session. It requires excellent organization and flexibility, the fixing of teachers’ dates with the students’ requests and the availability of the halls where the evaluation is to take place. Accommodating all these aspects is a tough challenge for those coordinating the teaching process in a given department.

The university runs a continuous examination session, comprising a period of one or more years of study, during which the exams can be taken at any date. A regular two-week exam session, held directly after the end of the term for all subjects, is unworkable here, not least for organizational reasons. Such a session is hardly feasible, especially for students of medical programs, where groups are enormous.

## 7. Traditional Examination vs. an E-Exam on an E-Learning Platform

Table 1 presents a comparison of the organization and implementation of knowledge evaluation, using a traditional paper version and an online evaluation. This is a complement to earlier classifications and comparisons, compiled at Poznan University of Medical Sciences in the years 2009–2013 [19], using the example of courses in pathophysiology, medical didactics and andragogy, biostatistics, mathematics or information technology, for different study programs.

Requirements that have to be met to conduct a computer-based evaluation of knowledge on an e-learning platform include:

1. Information technology facilities in the university. An increasing number of universities are now equipped with advanced facilities such as examination centers, medical simulation centers, libraries and computer labs for the Chair of Computer Science, fitted with a sufficient number of computers. An online exam can be held on the school’s e-learning platform. Another solution is to deploy smaller seminar rooms on the campus, each equipped with 20–25 computers, where online exams can be held with the support of administrative or technical-engineering staff. Benchmark tests throughout the course are run in classrooms where desktop computers or laptops can easily be installed without changing the arrangement or intended use of the room. To optimize the effective use of computer rooms and help examinees schedule their tests more conveniently, different exams can be administered in one place at one time. Some students can make their first attempt, others can retake, and those with an individual organization of studies might take their summative credit test.
2. Information technology facilities of the participants of the evaluation. Currently, every student has a few electronic devices with Internet access at their disposal. Consequently, online evaluation during the course can be conducted using students’ own equipment.
3. Testing out of school. According to an ordinance of the Minister of Science and Higher Education [49], an examination can be held outside of school under conditions that allow for supervision and recording of the exam.
4. Support from the experienced. The departments conducting online evaluation should receive technical support from the IT units responsible for the computer infrastructure of the university. The help provided by experienced teams, which develop and implement e-learning tools or educational research technologies, is vital [18,35,45,57]. These tasks are assigned to university e-learning centers, specialized research units, research departments or other larger academic departments, where e-learning specialists are employed part-time or for the duration of a project, supporting knowledge evaluation processes.

## 8. The Amount of Time Devoted to Preparing a Computer-Based Exam, on the School Premises, Compared to Its Traditional (Paper-Based) Version

Table 2 presents a sample application of the above comparison, including the analysis of the work time and tasks necessary to evaluate the basic science knowledge (pathophysiology) of 333 students attending the course on the school premises. The set comprises one final exam and three benchmark tests throughout the course: four tests in total. Benchmark tests are scheduled to be taken on predetermined dates during the classes. For some of the tasks, the amount of time is difficult to estimate, so they are described without a determined duration. However, staff members are able to state duration on an individual basis depending on the department in which they work.

## 9. The Analysis of the Amount of Time Needed to Create an Examination Question Bank

The creation of an electronic database of questions used for exams and credit tests consists of two stages of work, carried out by teachers alone or teachers supported by administrative or technical-engineering staff. The preparation of questions involves the time spent working on the actual substantive content and then the time needed to save them in the QTI format. This is a standard format used in computer-based knowledge evaluation held on e-learning platforms [19,20].

Studies conducted in the years 2015–2016 [58] demonstrated that teacher working time devoted to writing 20 pathophysiology test questions, with 4–5 answer choices in an electronic form, varied between 40 and 150 min. Therefore, the time needed by pathophysiology teachers to develop a bank of 200 questions is 18.5 h of work and an additional 8 h to export the questions in QTI format. This is performed by entering the questions from a document created by the author (teacher), followed by parameterization. This relates to, for example, answer keys, the random selection of answers, ways of displaying the question on the screen, or a random selection of questions from the bank. This work is performed once, and it serves its purpose for many years in the future.

Preparing a question bank of 200 questions for use in e-testing on an e-learning platform thus takes 26.5 h of work time. The analysis of the results in the above table for a computer-based test shows a calculated work time of 6.7 h, plus 26.5 h to create a question bank in QTI standard format. The summative result is 33.2 h. Traditional testing takes longer, approximately 72.2 h. This is the result of the analysis of the testing process presented in Table 2, plus the time spent composing the actual test questions (item 3, Table 2). We assume that 200 questions have to be developed for the paper-based test versions, which takes teachers about 18.5 h. Work time required in the case of a traditional test amounts to 90.7 h. Comparing the conventional form with the computer-based form, we can conclude that the latter is much more effective and beneficial for the educational institution, as it takes 37% of the time necessary to conduct a traditional form of testing. The time saved can be allotted to other teaching assignments, such as expanding the question bank at any time that they see fit.

The substantive content, depending on the nature of the subject, has a predicted lifespan of from 5 to 7 years from teaching the course [19,58,59]. The amount of time required to create an electronic question bank in the first year of e-testing and evaluation is more significant than that required for paper-based test versions, but it is spread over 5 to 7 years of use. The obtained values then have to be divided by at least 5, which provides the real hourly workload needed to develop an electronic question bank for a given academic year.

The size of the question bank developed for a given unit should depend on the number of groups, in which credit tests are administered as well as the number of course editions over one academic year. It is also important that a few test/exam dates are available per attempt, which is typical of the continuous examination session. The more students, test dates and course editions there are, the larger the base should be, to ensure an objective evaluation of student knowledge.

The amount of time required to expand the question bank in a database was also analyzed, and the results were calculated with regard to the work time needed for its development, along with the work time needed to prepare and carry out the testing process, comparing paper- and computer-based forms (Table 3).

Examining the results obtained in Table 3, we can see that, in the case of a question bank comprising 1600 test questions, the amount of time needed to organize and prepare the process of knowledge evaluation is similar for both paper-based and computer-based testing. When the number is increased to 2000 questions, the computer-based form requires more time than the traditional version. Work time is longer by 14.5 h, which is an increase in time of 5.6% compared with the traditional form. Plans to develop a base of 3000 questions or even 5000 questions leads these values to rise to 15.6%. This is, respectively, 54.5 h of work time for a 3000-question database and 134.5 h for a 5000-question database, which is the extra time required in comparison with the traditional form of testing.

Therefore, it seems legitimate to ask whether computer-based evaluation, which requires a database of over 1000 questions, is, in fact, as useful as previous studies have suggested.

To answer this question, other variables of the evaluation process must be analyzed, which influence the development of a question bank. These include a deadline for composing new questions, the number of teachers involved in the task and their IT competencies, and cooperation between units implementing e-evaluation. These aspects make it apparent that the traditional, paper-based form has severe limitations and are less useful when conducted on large groups of students, despite the reduced work time. When these aspects are added to the workload and organization time of the computer-based form, the difference is leveled for the excess time in the case of databases of over 1000 questions.

### 9.1. Time Pressure, Question Reusability

The sets of questions composed for paper-based tests form a base, which has to contain different or updated questions in every exam session. Paper-based test versions used with large groups of students in a given academic year are quickly known, so in order for them to be used again in the next academic year, they have to be revised and adjusted, which is as time-consuming as composing new questions. The work of writing questions for traditional tests has to be completed every year, and the deadline is determined by the pre-established schedule of tests. Consequently, the work is completed under time pressure, irrespective of the other assignments that teachers may have. That is why the development of question banks is sometimes abandoned, or too few questions are provided for the evaluation to be conducted properly. This stems from the ease of making the questions banks public, or a lack of randomness in test versions. For computer-based evaluation, the bank of questions can be enlarged as they can be supplied at any given time in the academic year, and combined with other activities. Inspiration for a valuable question could be derived from a discussion with students during a lecture or a clinical case study in a seminar. New test questions often appear as a result of the analysis of students’ work on the e-learning platform or their self-test scores. Then, the teacher enters the new questions in the QTI format to the database at a convenient time. The work is calmer and more thoughtful, which translates into valuable testing material, which will serve well in the verification of learning outcomes.

### 9.2. Cooperation between Units

It is rare for a unit to have a question bank of over 1000 questions. Composing such a large number of questions is a challenge in terms of time expenditure and content-related effort. To support the process of developing substantial content, a collaboration of a team of experts from a given unit or a whole school would be advantageous, as the time devoted to creating questions would be spread. Such a question bank saved in the international QTI standard format can easily be relocated to another e-learning platform, which implements computer-based evaluation standards. This, in turn, allows for different universities to share their databases, which naturally enlarges the pool of proven test questions. As a result, the time needed to develop questions for a single unit, calculated in Table 3, is significantly reduced. Such resources are invaluable to units collaborating in their creation for particular courses whose learning outcomes are the same in respective institutions. Such cooperation between teams of experts enables the workload to be significantly reduced. Writing exam questions is a complex and difficult process, so databases of over 1000 questions are an asset for many years to come, and clearly worth investing in.

### 9.3. A Further Period of Use

A question bank of the right size gradually reduces the amount of time necessary for creating substantive content and restricts the work to revising and updating the questions. This is not as time-intensive, and levels the excess time seen inn Table 3 for pools containing over 1000 questions. In the case of paper-based exams, the amount of time taken to develop the substantive content of questions is always the same, which is a strain for teachers.

### 9.4. Experience Backed by Statistics

Another important aspect concerns the analysis of the usefulness of test questions in the utilized database. Keeping the statistics and assessment of the question bank after the conducted evaluation in a given year seems to be a necessity. It allows the user to investigate the content, paying attention to the elimination of flawed test questions, ones that did not work or those at the wrong level of difficulty. It also serves to objectively analyze suggestions from students, who can express their reservations about questions after the exam or credit test. Questions should be thoroughly verified, with an emphasis on the scores achieved by all students taking the test. This will contribute to a reduction in the work time needed to supply new questions in future, and will definitely shorten the time spent on the substantive content of questions for banks containing over 1000 items.

### 9.5. ICT Competences of the Teaching Staff

A computer-based evaluation of student knowledge encourages the development of ICT competences in its participants, both students and teachers. Online testing will help the teachers improve their computer proficiency and develop their competences in this field, which will also contribute to a reduction in the work time needed in future concerning the conversion of questions to the QTI standard.

### 9.6. Summary of Work Time Analysis

The calculations made for databases of over 1000 questions demonstrate a longer work time needed for computer-based testing than paper-based testing; however, in the long term, the overall workload for a unit is reduced. It can thus be concluded that the electronic form is more advantageous and efficient than the traditional form.

Determining the labour cost and time involved in the process of knowledge evaluation in a particular teaching unit in one academic year must also consider the gains derived from the switch to an automated process. These include exempting assistants, administrative and technical-engineering staff from organizational duties connected with preparing and implementing the evaluation. The time devoted to preparing paper-based versions of tests, marking them using a template and archiving the results can be saved, and invested in developing their ICT competences. Their work on the e-learning platform will become more proficient and guarantee support to the authors of test questions in the creation and updating of items on the platform.

## 10. Discussion

The research was performed at the Department of Pathophysiology in cooperation with the Department of Computer Science and Statistics of Poznan University of Medical Sciences, Poland (PUMS). Academic teachers attended the research, as well as technical and administrative staff, and also e-learning experts. In 2009, this team introduced an e-learning portal for the entire university, further conducting its own research on the effectiveness and optimization of medical e-learning.

The research presented in the article was conducted by the Department of Pathophysiology, Poznan University of Medical Sciences, Poland on the ESTUDENT portal for remote education, which is an installation of the OLAT applications developed by the University of Zurich under an open-source license. The ESTUDENT portal is a proprietary LCMS application adapted to e-learning in the field of pathophysiology.

The described analysis of working time and the organization of electronic knowledge evaluation was carried out using an example of a large year of students in their second year of medicine in the preclinical subject of pathophysiology.

The working time of the analyzed knowledge evaluation through the e-learning portal is about 10% of the working time needed to carry out the evaluation in a traditional way. Electronic knowledge testing requires a greater amount of work time in the first year of application, due to the preparation of a larger database of questions compared to the number of questions required for the evaluation of knowledge conducted in the traditional (paper) version. However, teachers’ working time is spread over 5–7 years of using the electronic question base. As part of the research, an analysis of the working time of building the database from 200 to 5000 test questions for the evaluation of knowledge in e-learning and the evaluation carried out in the traditional version was performed.

The data presented in the article are the result of pioneering research conducted by the authors in the field of the evaluation of preclinical knowledge of very numerous generations of medical students using the e-learning portal, which was carried out in 2009–2019. The described electronic realization used the example of the academic year of 2018–2019, when 333 students were studying medicine. On the basis of our own research, in 2015–2016, the time spent by teachers on exam questions was measured for those participating in traditional education and the e-learning portal. To conduct this research, the same team of academic teachers must participate, and the same conditions must be met for the implementation of both traditional knowledge evaluation and evaluation using e-learning methods.

The confirmation of the usefulness of e-learning in medical education is in the comparison of the benefits and limitations of the electronic evaluation of knowledge and the didactic process and evaluation using the traditional implementation. The work contains such analyses, also indicating the different stages of conducting these components of education in the e-learning portal. An important element of the research was the analysis of the work time needed for the preparation and implementation of electronic knowledge evaluation. The results clearly indicate the advantage of e-learning over traditional organization in terms of the implementation of examinations and surveys.

## 11. Summary of Study Results

The application of e-learning for computer-based knowledge evaluation and optimizing the administration of exams for students of medical sciences should be considered in two categories: from the perspective of the providers of the evaluation process, that is, the teaching unit, and from the recipients of the evaluation process, that is, the students.

The advantages to computer-based evaluation providers, that is, the teaching unit, include:

1. Automated test marking and information about the result (course credits, passing an exam).
2. Exporting test scores as a spreadsheet with the possibility of importing scores to a statistical package for quick analysis of the obtained data.
3. Full archiving of all evaluation results, including practice tests (self-tests) and papers submitted as project work or group work during the course or as a credit requirement.
4. Online access to current and archived results of evaluations, broken down by group, date or type of test, with full information about the test results for each student.
5. Web-based questionnaire administration. The excess time during the evaluation can be used to administer course evaluation questionnaires or study surveys. This encourages student participation in research projects and ensures a high questionnaire return rate, which cannot be said for paper questionnaire distribution.
6. Developing a question bank without time or deadline pressure, except for the first year of e-evaluation, which requires a heavier workload and more time for preparation. An extensive database is more cost-effective in the long term.
7. Random selection of test questions from a large database with a random order of possible answers, providing a more objective assessment of a student’s knowledge.
8. An opportunity to use questions based on graphic or multimedia elements. In paper-based evaluation, such possibilities cannot be used efficiently; an exam could be held in a room with Internet access and projector but, in the case of a large group of examinees, this is organizationally difficult.
9. Investment in the development of the staff’s ICT competences, resulting in a higher proficiency in work on the e-learning platform and its full application in the organization and realization of the didactic process. The time saved can be devoted to composing new test questions.

The advantages to computer-based evaluation recipients, that is, the students, include:

1. Test results are delivered immediately after the test. In the case of practice tests, the student receives detailed results accompanied by correct answers, explanations and hints.
2. Access to all evaluation results from the course in one place, on the e-learning platform.
3. Automated and individual control of each test-writing time.
4. Independence of students’ work during a test or examination. Each examinee has an individual, random selection of questions, drawn from an extensive database, with a random order of answer choices and a time limit depending on the need and test parameters.
5. Comprehensive communication regarding the organization of evaluation via the e-learning platform. This includes enrollment for test dates with the possibility of cancelling and changing them, without the necessity to contact the unit or group representatives, who often compile lists of students and deliver them to teachers. It also includes the submission of complaints or remarks concerning test questions online after the test.
6. Computer-based evaluation saves students’ time and improves their participation. The time saved can be dedicated to additional study and revision before the exam.

## 12. Limitations

The authors’ research indicates several factors common to the evaluation of knowledge in large groups of students completing a subject at one time, and the factors significantly influencing the optimization of this process. This includes the labor-intensive preparation of databases with questions, the competence level of suppliers (teachers) and recipients (students) of knowledge in the field of e-learning technologies, ensuring conditions for independent work (parameterization of tests or examination rooms), archiving results or the speed of feedback after evaluation knowledge.

Certain elements of this process are changeable and difficult to standardize, depending on the university’s IT infrastructure. The differences may be related to the type of e-learning application or technical service support, which can be expanded on, and is at the full disposal of the candidates (university-wide center) or available locally (unit’s own resources).

In order to optimize the implementation of the process, both traditional and e-learning variants should be carefully analyzed with the same human team, as shown in the diagram from the university, paying attention to its individual conditions (limitations, possibilities during a pandemic) and the specificity of issues. The final calculations may, therefore, differ slightly from those presented in the article. The analysis presented by the authors, as an example, indicates the superiority of the evaluation using e-learning technologies compared to the traditional evaluation. It proposes solutions to the optimal direction of this process, paying attention, for example, to cooperation between units, and sharing resources that will minimize the time spent working on question databases. The presented analysis is typical of universities working on open-source portals with limited funding, which is common in Eastern and Central Europe. It allows for a successful, remote execution, quickly, in one’s own unit, over times such as the SARS-CoV-2 pandemic.

There are no complete, detailed analyses with the work time for all stages of work and the organization of medical evaluation for groups of more than 300 students. In order to be perform such analyses, the same team of academic teachers should be involved, and the same requirements should be met in the implementation of both traditional and remote application knowledge evaluation. Research also requires time and experience in the field of e-learning, which significantly affects the effectiveness of the process. There is no well-established educational theory for e-medical education, as remote methods contain parameters that are not known in traditional medical education. Research and discussion on the standardization of e-education and the development of patterns into which medical universities and schools are forced by the pandemic, testing of existing solutions, indications of limitations and addition of new variables are necessary in the important process of evaluation of the knowledge of medical students, who will become doctors (physicians).

## 13. Conclusions

Replacing the traditional form of organizing and implementing knowledge evaluation with a web-based equivalent on an e-learning platform optimizes the whole didactic process, not only for the unit carrying this out but, above all, for students. Due to this innovation, course participants have the opportunity to take full advantage of all the technological solutions that e-learning provides, with an implementation that can start from computer-based evaluation. The process of optimization through e-learning should become a natural part of the didactic process, conducted in every subject at all types of higher education institutions, including medical universities. The obtained results encourage their implementation, considering the nature and conditions of medical training, which is a key program in medical universities.

## Figures and Tables

**Table 1:** A comparison of tasks necessary for the implementation of student evaluation.

No.	Task	Method of Testing	
		Paper-Based	Computer-Based
1.	Preparing test questions	For a single exam	Question bank sufficient for a few years of testing
2.	A few sets of questions for a test	Preparing new versions of questions is necessary	A unique collection of questions for each student from the existing question bank
3.	Preparing questions for the retake	New versions necessary	Preparation of new questions; unnecessary questions are drawn from the existing question bank
4.	Graphic- and multimedia-based questions	Difficult and rarely used. The projector is available in halls with Internet access. Difficult to manage with large groups of examinees.	Easy to manage and increasingly used.
5.	Copying and distributing	Printing paper version. Confidentiality necessary	None. Confidentiality unnecessary
6.	Duration of the test	Difficult to enforce a timeframe with large groups of examinees.	Automated and individual time control for each student
7.	Independence of work	Dubious. A few (2–3) versions of the test with the same order of questions and answers. Difficult to manage with large groups of examinees.	Independent work of the student. Each student gets a (randomly selected) set of questions with a random order of answers.
8.	Assessment	Necessary. Work after the exam	None. Automated instant assessment on the platform
9.	Exam results	After some time. Within five working days	Immediately after the test
10.	Issuing the results	Necessary. Sending the results by e-mail or entering an e-form of student achievement (virtual student office)	None. Automated information in student account on the platform
11.	Exam archiving	Necessary. Labelled sets of tests must be stored in an indicated place in an arranged class/group order	None. Automated, with access to tests by any search criteria for every class
12.	Course evaluation/participation in evaluation surveys	Rarely used. Requires additional preparation (printing) and organization of the exam.	Easy to administer. Fills the free time before or after taking the exam.
13.	Exam paper score review (assessment feedback) for students and teachers	Appointment in the storage room requires searching for the exam paper.	Instant review in any place with Internet access.

**Table 2:** The amount of time devoted to preparing and administering knowledge evaluation using traditional paper-based tests vs. online tests on an e-learning platform.

No.	Task	Method of Testing	
		Paper-Based	Computer-Based
1.	People involved in the evaluation	T: teachers and A: assistants, administrative staff or technical-engineering staff	A: assistants, administrative staff or technical-engineering staff and T: teachers.
2.	Preparing test versions	T. prepares and sends the A. 2–3 electronic versions of tests by e-mail	0 min. Question bank sufficient for a few years. The time needed to create it currently under analysis
3.	Copying and printing	(60–120 min) A’s work with four tests. Preparation of tests divided by group, printing paper versions. Assumed time: 15–30 min per test.	0 min. None
4.	Registration for an exam.	A. Collecting lists of students, coordinated by A. via e-mail or in person with group representatives.	Online, on the platform. The student registers with a convenient date, time, place. The choice can be cancelled.
5.	Order of questions and answer choices in the test	The same, 2–3 versions of the test. Independence of students’ work difficult to oversee.	Different, random set of questions and answers—multiple versions. Independent student work
6.	The Time required to administer one exam on the premises. Benchmark tests are held during classes.	(100–200 min) T and/or A: two supervisors simultaneously 100 min each (auditorium hall), or in two rounds on one day with two supervisors × 100 min = 200 min (lecture hall).	(400 min) A and/or T: four supervisors in four computer rooms simultaneously. Exam carried out in four rounds at four times on one day: 4 × 100 min = 400 min. Department can accommodate 84 students per round.
7.	Assessment. (marking students’ work).	(3996 min) A: We assume 3 min. Is necessary to mark one student paper with a template. 333 papers × 4 tests × 3 min = 3996 min = 66.6 h equals 8 working days One test: 333 papers × 3 min = 999 min = 16.7 h (approx. 2 working days).	0 min.
8.	Test results	Students receive the results a few days later—up to 5 working days.	The student knows the result immediately after the test.
9.	Marking (grading) errors	(30 min) A: Highly probable. Time is required for re-marking, counting the points again and sending explanations to students.	None.
10.	Test result delivery	(44.4 min) A: Entering the results to an e-form of student achievement (virtual student office) and notifying students. We assume a required time of 2 s per result. 333 papers per test × 2 s = 666 s, with four tests this amounts to 4 × 666 s = 2664 s = 44.4 min. There is a likelihood of committing errors while entering the results.	0 min.
11.	Archiving test papers on the premises	A. 333 papers per test For 4 tests, 1332 papers are stored in cabinets on the premises.	None. All works are available online—see the example below.
12.	Calculating the average score for crediting after three benchmark tests	(15–30 min) A. Advanced use of spreadsheets required. Result analysis concerning retakes and preparing a list of people who failed the course.	0 min. Automated scores on the platform. Result lists can be imported as an .xls file.
13.	Access to current and past results and papers	A. Searching for paper-based tests in department archives and/or in files on a computer disc—is time-consuming	A/T. Full access on the platform (example—figure)
14.	Result availability for students	None.	Yes. All test results are available in one place on the platform (example—figure)
15.	Course evaluation/study surveys	A. Difficult to conduct—rarely practised. (1) Survey/questionnaire printout necessary. (2) Significant amount of time required to enter hand-written answers in the file. (3) Poor hand-writing legibility.	Frequently practised—guarantees close to 100% student participation. (1) Automated process. (2) 0 min. time required to enter hand-written answers to the file. (3) Everything is instantly recorded in a file on the platform, ready to be imported to an external data storage device.
Total of work time to preparation and implementation of knowledge evaluation:		4245.4–4420.4 min. equals 70.8–73.7 h Assumed average = 72.2 h	400 min = 6.7 h
Recapitulation of significant aspects of the organization of knowledge evaluation:		(1) printout of large numbers of test papers, (2) required space for archiving thousands of test papers, (3) score review possible only on the premises, during the school’s working time, (4) T. needs support from A./staff for distributing, assessing and archiving tests, (5) T. creates a few test versions for every course edition. Work is demanding, taking place under time and deadline pressure.	(1) more people physically involved in conducting the exam, (2) access to computer rooms, (3) labour-intensive creation of question bank in the first year of testing, which is a capital for the future. Subsequent editions of the course require minor corrections, updating the database. New questions created by T. are continually supplied over the whole year, without the time and deadline pressure.

Symbol *h* in the table stands for *one hour*—60 min.

**Table 3:** The analysis of work time devoted to developing a question bank for paper-based and computer-based knowledge evaluation.

Number of Questions in the Base	Work Time in Hours (60 min) [h]			
	Developing a Question Bank (Item 2 in Table 2)		Organization and Implementation of the Knowledge Evaluation Process (All Items from Table 2)	
	Paper-Based Test ^I^	Computer-Based Test ^III^	Traditional Testing ^II^	Computer-Based Testing ^IV^
1 × 200 = 200	18.5	26.5	90.7	33.2
2 × 200 = 400	37	53	109.2	59.7
4 × 200 = 800	74	106	146.2	112.7
5 × 200 = 1000	92.5	132.5	164.7	139.2
8 × 200 = 1600	148	212	220.2	218.7
10 × 200 = 2000	185	265	257.2	271.7
30 × 200 = 3000	277.5	397.5	349.7	404.2
50 × 200 = 5000	462.5	662.5	534.7	669.2

^I^ time for developing the content of 200 questions—18.5 h; ^II^ the result of adding the amount of time from the *paper-based test* column and the time calculated in Table 2: 72.2 h; ^III^ time for composing 200 questions and saving them in QTI format—26.5 h; ^IV^ the result of adding the amount of time from the *computer-based test* column and the time calculated in Table 2: 6.7 h.

## Data Availability

Department of Pathophysiology, Poznan University of Medical Sciences, Poland; www.estudent.ump.edu.pl (accessed on 29 August 2021).
